# Development and evaluation of a milk yield-based indexing framework for mastitis monitoring in dairy cows

**DOI:** 10.5713/ab.260447

**Published:** 2026-09-01

**Authors:** Jae-Woo Song, Woo-Kyung Lee, Hyunjin Cho, Mingyung Lee, Seung-Hyun Lee, Seongwon Seo, Wang-Hee Lee

**Affiliations:** 1 Department of Smart Agriculture Systems Machinery Engineering, Chungnam National University, Daejeon, Korea; 2 Department of Smart Agriculture Systems, Chungnam National University, Daejeon, Korea; 3 Division of Animal and Dairy Sciences, Chungnam National University, Daejeon, Korea

**Keywords:** Dairy Cattle, Mastitis Alert, Milk Yield Indexing, Smart Dairy Farming

## Abstract

**Objective:**

Mastitis is a major disease affecting dairy cows that requires timely detection to enable efficient management and to reduce substantial economic losses. In this study, we developed a practical mastitis monitoring framework based exclusively on daily milk yield, a routinely collected and noninvasive variable readily available from automated milk-recording systems.

**Methods:**

Daily milk yield records (n = 121,692) from 555 Holstein cows on four Korean dairy farms were analyzed. Lactations were reconstructed, and mastitis datasets were matched 1:1 with non-mastitis controls by farm, date, and days in milk. After completeness screening, 63 matched pairs were divided into development (44 pairs) and test datasets (19 pairs). Expected yield was estimated using exponential smoothing and a modified Wilmink equation. The index combined milk yield reduction, a day-specific 90% lower prediction threshold, and consecutive negative deviations. Index changes were compared across 7-, 9-, and 11-day intervals, and alert rules based on index level, relative decline, or both were optimized in the development dataset and evaluated in the test dataset.

**Results:**

In mastitis datasets, mean index scores gradually decreased from the pre-diagnosis to post-diagnosis intervals across all window sizes, with significant differences among all three intervals (p<0.05). No significant interval-dependent differences were observed in matched non-mastitis datasets. In the development dataset, the highest F1-score was 0.800 at day +7 using a 3-day evaluation window and a 5% relative-decline rule. In the test dataset, the best and most balanced performance was observed from days +4 to +6, with accuracy, sensitivity, specificity, and F1-score all reaching 0.790.

**Conclusion:**

Persistent deviations from expected daily milk production patterns can be converted into an interpretable alert signal for mastitis-associated changes. The proposed framework may support practical and noninvasive identification of cows requiring closer observation or confirmatory testing without additional sensors or invasive sampling.

## INTRODUCTION

Mastitis is a highly prevalent and economically important disease in dairy cattle, causing substantial losses to the dairy industry through reduced milk yield, impaired milk quality, increased treatment costs, and premature culling [1,2]. Because mastitis directly disrupts mammary gland function and can rapidly progress and spread within the herd, timely identification of affected cows is essential for effective herd management and appropriate intervention [3]. However, in commercial dairy systems, frequent monitoring is often impractical when detection primarily depends on visual inspection or periodic confirmatory testing. This challenge is particularly evident in large-scale herds, where repeated individual-level assessments are constrained by labor and cost requirements [4,5]. Consequently, mastitis may not be detected during the early stages of disease progression, potentially delaying treatment and increasing the risk of production losses and disease transmission.

Conventional mastitis detection methods include visual examination, the California mastitis test (CMT), somatic cell count-based assessment, and other physiological or sensor-based indicators [1]. Although these methods provide valuable diagnostic information, they require manual inspection, additional sampling, laboratory analysis, or specialized equipment. These requirements may limit their routine application as continuous early warning systems on large-scale dairy farms, where labor efficiency, cost-effectiveness, and ease of implementation are critical considerations. To improve automated mastitis monitoring, previous studies have evaluated physiological and sensor-derived variables, including electrical conductivity, activity, and rumination time [6–9], as well as image-based approaches such as infrared thermography and thermal imaging of udder surface temperature [10,11]. Multivariable approaches using data from automatic milking systems have also been investigated, with some studies applying statistical or machine learning methods to combine multiple indicators for clinical mastitis prediction [8,12]. Despite their potential, many of these approaches depend on additional sensors, quarter-level measurements, or system-specific multivariable data streams, which may restrict their practical implementation and scalability across farms. Therefore, there remains a need for simple, practical, and noninvasive monitoring approaches that can be implemented using routinely collected farm data.

Daily milk yield, automatically recorded in many modern milking systems, represents a readily available source of information for mastitis monitoring in the field [13]. Previous studies using automatic milking systems have reported that changes in milk yield and electrical conductivity can occur before the onset of subclinical mastitis, supporting the value of production-related indicators for disease monitoring [14]. Because mastitis disrupts mammary gland function and impairs milk synthesis and secretion, disease onset is frequently linked to measurable alterations in milk production dynamics, particularly sustained declines in yield. This supports the biological relevance of milk yield as a potential indicator of abnormal health conditions in dairy cows [15]. Previous studies have shown that mastitis events negatively affect milk yield, particularly during early lactation. In addition, pathogen-specific clinical mastitis has been associated with considerable milk losses and compositional changes in affected cows [16,17]. However, absolute reductions in daily milk yield are challenging to interpret as direct indicators of mastitis because milk production is also influenced by numerous biological and management-related factors, including baseline productivity, parity, lactation stage, feeding conditions, milking interval, and environmental stress [18,19]. Moreover, short-term fluctuations in milk yield can arise even in the absence of disease, making it difficult to distinguish transient variation from biologically meaningful abnormalities [20]. Therefore, an effective monitoring approach should evaluate milk yield in relation to its expected temporal pattern and capture persistent downward trends rather than isolated reductions in production.

Accordingly, in this study, we developed a daily milk yield-based indexing framework that integrates predicted yield, smoothed temporal trends, confidence-interval-based criteria, and consecutive negative deviations between smoothed and predicted values. The proposed framework was designed to convert daily milk production patterns into quantitative and interpretable scores suitable for practical on-farm monitoring. Because the timing and duration of the evaluation period may substantially affect the identification of mastitis-associated changes, multiple temporal windows were evaluated to characterize changes in index scores and optimize mastitis alert rules. Consequently, our objectives were to establish practical indexing criteria for daily milk yield evaluation, characterize mastitis-associated temporal changes in index scores, and optimize and validate milk yield-based mastitis alert rules under commercial farm conditions.

## MATERIALS AND METHODS

### Data collection and mastitis diagnosis

Data were collected from Holstein dairy cows raised on four farms in Cheonan-si, Dangjin-si, and Asan-si, Chungcheongnam-do, South Korea, between January 2020 and August 2021. Farm 1 was located in Cheonan-si and used the Afimilk system, Farm 2 was located in Dangjin-si and used the Afimilk system, and Farms 3 and 4 were located in Asan-si and used the DeLaval and BouMatic systems, respectively. Milk yields were measured in herringbone milking parlours equipped with electronic milk meters and individual cow identification systems. All records were automatically stored and managed using automated milk-recording and herd-management software. The acquired dataset included daily milk yield and mastitis incidence records categorized according to three diagnostic criteria: the inline clot filter method, CMT, and visual observation. The acquired dataset included daily milk yield and mastitis incidence records categorized by three diagnostic methods: (1) the clot filter method, which detects physical abnormalities by screening for milk clots using inline filters [21]; (2) the CMT, a rapid on-site screening method for estimating somatic cell counts based on reagent-induced viscosity changes [22]; and (3) visual observation involving expert clinical assessment of udder abnormalities [23]. Multiple diagnostic methods were available for mastitis labeling, and more than one positive label could be recorded for the same cow on the same date. Mastitis events were defined when at least one diagnostic method yielded a recorded positive result, and overlapping positive records for the same cow on the same date were treated as a single event.

### Data structure and preprocessing

A total of 121,692 daily milk yield records were obtained from 555 dairy cows across four farms. Because information on calving dates and days in milk (DIM) was unavailable, individual lactation periods were reconstructed from the longitudinal milk yield records. Records with zero or negative milk yield were considered invalid and excluded. A new lactation was defined when milk recording resumed after at least 40 consecutive days without a valid milk yield record. The first valid milking day within each identified lactation was designated as DIM 1. Records that could not be assigned to an identified lactation period were excluded from subsequent analyses. In total, 24,839 records with zero or negative milk yield and 23,764 records outside the identified lactation periods were removed.

Following preprocessing, 650 lactation datasets were identified, including 119 with at least one recorded mastitis diagnosis and 529 without mastitis. Mastitis was diagnosed using three on-farm methods (CLOT, CMT, and VO). Because multiple diagnostic methods could be recorded for the same cow on the same date, a unique mastitis event was defined by farm, cow, and calendar date, irrespective of the number of positive diagnostic labels. Consequently, the 119 mastitis lactation datasets contained 177 unique mastitis events, comprising 69 CLOT, 71 CMT, and 65 VO positive labels, with 28 events having multiple concurrent diagnostic labels (Table 1). Among the mastitis datasets, 90 satisfied the eligibility criteria for matching. Each was matched 1:1 with a non-mastitis control according to farm, calendar date, and DIM, using an initial matching criterion of ±10 DIM that was extended to ±15 DIM when necessary. This process yielded 72 matched pairs (63 within ±10 DIM and nine within ±15 DIM; mean absolute DIM difference = 3.97 days). Because each farm used a single milk-recording system, matching within farms ensured that both datasets in each pair originated from the same recording system. Following assessment of data completeness over the index-analysis period (day −11 to day +11), 63 matched pairs were retained for the final analysis. These comprised 63 mastitis and 63 non-mastitis lactation datasets, representing 63 unique mastitis events. The final mastitis dataset included 27 CLOT, 18 CMT, and 26 VO positive labels, with eight events containing multiple concurrent diagnostic labels.

The 63 matched pairs were divided at the pair level into development (44 pairs) and test (19 pairs) datasets while preserving the development-to-test proportion within each farm. The development dataset was used for optimization of the mastitis alert rules, and the test dataset was reserved for independent evaluation. Because parity information was unavailable and all datasets originated from the same four farms, parity effects could not be assessed and the test dataset represented internal rather than external validation.

### Algorithm development for milk yield indexing

To quantitatively evaluate daily milk yield for mastitis monitoring, reductions in milk yield were incorporated into an indexing framework based on predicted milk production. Milk yield was predicted using exponential smoothing of historical milk yield data. The prediction model utilized a regression framework grounded in the modified Wilmink equation (Eq. 1), a combination previously recognized for its superior performance in milk yield prediction [24].


(1) $$y=a+be^{-0.05t}+ct^{2}+dt$$

where y is the daily milk yield, and t is the days of milk.

Based on the predicted milk yield, two scoring approaches were developed. The first approach utilized two criteria derived from the cumulative milk yield dataset: the difference between the predicted milk yield (calculated using cumulative data) and exponentially smoothed value and the reduction rate of the exponentially smoothed value relative to the predicted value (Eq. 2). The second approach extended the first method by incorporating a confidence-bound criterion derived from the cumulative dataset. A 90% confidence level was selected because it provided the highest sensitivity for detecting mastitis-associated milk yield declines. A daily record was classified as abnormal when the observed milk yield fell below the corresponding lower confidence bound (Eq. 3).


(2) $$Reduction\;rate=\frac{\left(Predicted\;value-Smoothed\;value\right)}{Predicted\;value}$$

(3) CI criterion=Predicted value on the corresponding day-1.645×SD

where CI is confidence interval; and SD is standard deviation of the predicted value.

The final index score was established through a multi-stage evaluation procedure. First, an initial score was calculated using the mean yield reduction over a 7-day rolling window preceding the evaluation date. Second, the score was adjusted downward based on the frequency of observations falling below the lower bound of the 90% CI. Finally, the score was weighted based on the difference between the exponentially smoothed and predicted values, with an additional penalty factor applied according to the number of consecutive negative deviations (smoothed value−predicted value) (Table 2).

### Interval-based evaluation of index score changes

To characterize temporal changes in the index score around mastitis diagnosis, interval-based analyses were performed using the development dataset. Three sequential intervals were defined relative to the recorded diagnosis date for mastitis datasets and the corresponding reference date for matched non-mastitis datasets: Interval 1 (pre-diagnosis), Interval 2 (centered on the diagnosis/reference date), and Interval 3 (post-diagnosis). Analyses were conducted using 7-, 9-, and 11-day windows. For the 7-day window, the intervals corresponded to days −7 to −1, −3 to +3, and +1 to +7, respectively; for the 9-day window, days −9 to −1, −4 to +4, and +1 to +9; and for the 11-day window, days −11 to −1, −5 to +5, and +1 to +11 (Figure 1).

A mean index score was calculated for each interval within each lactation dataset. Because the distributions of within-dataset differences did not satisfy the normality assumption, interval differences were evaluated separately for mastitis and matched non-mastitis datasets at each window size using the Friedman test, followed by Wilcoxon signed-rank tests with Holm adjustment for pairwise comparisons when significant (adjusted p<0.05) [25,26]. To further quantify temporal changes within individual lactations, signed (later interval − earlier interval) and absolute differences between interval mean index scores were calculated for the comparisons of Intervals 2 vs. 1, 3 vs. 1, and 3 vs. 2. Negative signed values indicated a lower index score in the later interval. The mean absolute difference and the standard deviation of the signed differences were used to summarize the magnitude and variability of temporal changes. All analyses were implemented in Python 3.9.13, and the framework was retrospectively applied to routinely collected daily milk yield records.

### Optimization and validation of mastitis alert rules

Mastitis alert rules were developed using the 44 matched pairs in the development dataset. A fixed four-day baseline interval (days −11 to −8 relative to the diagnosis date) was used to represent the pre-diagnosis index level. Evaluation windows of 3, 5, and 7 days were assessed, with the end day ranging from day 0 to day +11, resulting in 12 evaluation time points. The same relative-day definitions were applied to the matched non-mastitis datasets using their corresponding reference dates. For each combination of evaluation-window length and evaluation end day, the mean index score and its relative decline from the baseline interval were calculated. Three alert rules were evaluated: an index rule based on the mean index score, a ratio rule based on the relative decline, and a combined rule requiring both criteria. Candidate threshold values were optimized in the development dataset, and performance was assessed using accuracy, sensitivity, specificity, and F1-score. For each evaluation end day, the rule type, window length, and threshold combination yielding the highest F1-score was selected. The optimized rules were then applied without modification to the independent test dataset, and classification performance was evaluated using the same metrics.

## RESULTS

### Interval-based changes in index scores according to window size

Interval-based changes in index scores were evaluated using the development dataset comprising 44 mastitis and 44 matched non-mastitis datasets. In the mastitis group, mean index scores progressively decreased from Interval 1 (pre-diagnosis) to Interval 3 (post-diagnosis) across all window sizes, whereas no comparable trend was observed in the non-mastitis group, where mean scores remained relatively stable or increased slightly (Table 3). The magnitude of the decline between Intervals 1 and 3 increased modestly as the window size increased. In addition, the variability of index scores increased across intervals in the mastitis group but remained stable or decreased in the non-mastitis group.

Comparisons of interval mean scores further supported these findings (Table 4). Signed differences were consistently negative in the mastitis group, indicating progressive declines in index scores over time, whereas differences in the non-mastitis group were small and close to zero. Similarly, the mean absolute within-dataset differences were consistently larger in the mastitis group, particularly for comparisons involving Interval 3, indicating greater temporal changes in index scores following mastitis diagnosis.

Wilcoxon signed-rank tests with Holm adjustment confirmed significant differences among all three intervals in the mastitis group for the 7-, 9-, and 11-day windows (all adjusted p<0.05; Figure 2). In contrast, no significant interval-dependent differences were observed in the matched non-mastitis group at any window size.

### Optimization of detection timing using sliding-window analysis

Classification performance in the development dataset generally improved as the evaluation end day extended beyond the recorded diagnosis date (Figure 3; Supplement 1). Across all evaluation-window lengths, F1-scores were lowest when the evaluation ended on day 0 and increased as additional post-diagnosis records were included. The optimal rule configuration varied according to evaluation end day, although ratio rules based on a 5% decline from the baseline interval were selected most frequently after day 0 (Table 5). On day 0, the best-performing configuration was a 5-day index rule (F1-score = 0.537). At day +3, a 3-day ratio rule achieved an F1-score of 0.716. The highest performance in the development dataset was observed at day +7 using a 3-day ratio rule with a 5% decline threshold, yielding an accuracy of 0.796, sensitivity of 0.818, specificity of 0.773, and an F1-score of 0.800.

The optimized rules were subsequently applied to the independent test dataset without further modification (Table 6). Similar to the development dataset, classification performance improved as additional post-diagnosis records became available. On day 0, the selected 5-day index rule showed relatively low specificity (0.421), resulting in an F1-score of 0.571. Performance improved by day +3, where the 3-day ratio rule achieved an F1-score of 0.757. The highest and most balanced performance was observed from days +4 to +6, despite differences in the selected rule configurations, with accuracy, sensitivity, specificity, and F1-score all reaching 0.790. From days +7 to +11, sensitivity remained unchanged (0.790), whereas specificity decreased to 0.684, resulting in a slight reduction in overall performance. Overall, the independent test dataset showed the same general trend as the development dataset, although peak performance occurred earlier (days +4 to +6 rather than day +7).

## DISCUSSION

In this study, we established a milk yield-based indexing framework for mastitis alert generation and demonstrated that its classification performance depended on the timing and length of the evaluation window. In the development dataset, the highest F1-score was obtained at day +7 using a 3-day relative-decline rule, whereas the independent test dataset showed the highest and most balanced performance from days +4 to +6. These findings indicate that incorporating several days of milk yield records after the recorded diagnosis date provided more stable discrimination than evaluation on the diagnosis date alone, rather than supporting a single universally optimal temporal configuration. In addition, index scores consistently decreased across the sequential intervals in mastitis datasets for all evaluated window sizes, whereas no significant interval-dependent changes were observed in matched non-mastitis datasets. This finding further demonstrates that deviations from expected milk production patterns can serve as practical and noninvasive indicators of mastitis-associated production changes. A key aspect of the proposed approach is its reliance on a composite index rather than on raw milk yield reduction alone. Interpreting raw milk loss can be inherently challenging because daily yield is strongly influenced by factors, such as baseline productivity, parity, lactation stage, and short-term environmental variation [19,27]. In contrast, the proposed scoring framework integrated predicted yield, smoothed temporal trends, prediction-value-based CIs, and the number of consecutive deviations between smoothed and predicted values. This approach enables differentiation between transient fluctuations and persistent disease-associated declines, thereby providing a more standardized and biologically interpretable indicator of abnormal production dynamics than that provided by milk yield alone. The observed relationship between mastitis occurrence and decreasing index scores is biologically plausible because mastitis directly impairs mammary gland function and reduces milk synthesis [28]. Inflammatory responses in the udder disrupt secretory epithelial activity, impair milk letdown, and induce local tissue damage, all of which contribute to reduced milk yield [29,30]. In addition, systemic disease-related stress may alter nutrient partitioning and feeding behavior, thereby exacerbating milk yield loss [31]. Consequently, the decline captured by the proposed index reflects not only a statistical trend but also the physiological effects of mammary inflammation.

Temporal window length had different implications for characterizing index-score changes and optimizing mastitis alert performance. In the interval-based analysis, increasing the window size from 7 to 11 days generally increased the magnitude of the difference between the pre-diagnosis and post-diagnosis interval means in the mastitis group. This finding suggests that longer intervals more clearly summarized sustained milk yield reductions around the recorded diagnosis date. However, classification performance did not improve monotonically with evaluation-window length. The highest F1-score in the development dataset was obtained using a 3-day evaluation window ending on day +7, whereas the test dataset showed the highest and most balanced performance from days +4 to +6 using 3-, 5-, and 7-day windows, respectively. Therefore, the results do not support a single universally optimal window length. Instead, they indicate that the appropriate window depended on the evaluation end day and the type of alert rule applied. This pattern reflects the inherent trade-off associated with temporal aggregation of daily milk yield records. Shorter windows place greater emphasis on recent changes and may therefore preserve abrupt production declines, but they may also be more sensitive to transient day-to-day fluctuations. Conversely, longer windows may reduce the influence of short-term variation and better represent persistent reductions, while potentially averaging abrupt changes across a broader period. Daily milk yield can vary according to milking interval, feeding and management conditions, parity, lactation stage, and environmental stress, independent of disease status [32–34]. Moreover, the magnitude and temporal pattern of mastitis-associated milk loss can vary among cows and may persist or progressively manifest after diagnosis [28]. Accordingly, the improved performance observed when evaluation windows extended several days beyond the recorded diagnosis date may reflect both the continued expression of mastitis-associated production loss and the increased stability obtained by incorporating multiple daily records. The proposed framework should therefore be interpreted as a milk yield-based alert and monitoring approach rather than as a system that consistently identifies mastitis before clinical diagnosis.

The primary practical implication of this study is that routinely collected milk yield data can be converted into a cost-effective, noninvasive signal for mastitis monitoring. Rather than replacing definitive diagnostic methods, the proposed framework may function as an alert system that identifies cows requiring closer observation or confirmatory testing. This approach may be particularly useful on large commercial dairy farms, where frequent individual-level mastitis testing can be constrained by labor and cost. Because the framework applies predefined indexing and alert rules without requiring additional model training, it can be updated whenever new daily records become available. Combined with the automatic collection of daily milk yield data through existing milking and herd-management systems, this provides a structure suitable for timely on-farm monitoring without requiring additional invasive measurements or specialized sensors [35,36].

This study has several limitations. First, the final analysis included only 63 matched mastitis–non-mastitis pairs, and both the development and test datasets originated from the same four farms, limiting the evaluation to internal validation. Prospective validation in larger and more diverse dairy herds is therefore required to confirm the robustness, generalizability, and practical applicability of the proposed framework. In addition, direct cross-system calibration was not possible because each farm used a single milk-recording system, and system effects could not be separated from farm effects. Furthermore, agreement among the three diagnostic methods could not be formally evaluated because complete contemporaneous diagnostic records were unavailable, although the inclusion of multiple routine on-farm diagnostic methods may improve the practical relevance of the framework. Second, parity information and exact calving dates were unavailable. Consequently, lactation periods and DIM were inferred from milk yield records, and parity- and lactation-stage-specific effects were not incorporated into the alert rules despite their potential influence on milk production patterns [19]. Finally, differences in farm environments and herd-management practices may also have contributed to the observed variability. Despite these limitations, this study demonstrates the feasibility of converting routinely collected daily milk yield records into a standardized mastitis alert signal under commercial farm conditions. Future studies should prospectively evaluate the proposed framework across a larger number of farms and determine whether its integration with in-line milk sensors capable of measuring somatic cell count, electrical conductivity, and milk composition, including fat, protein, and lactose, as well as wearable activity and rumination monitors, can improve the timeliness, specificity, and overall reliability of mastitis alerts [37,38].

## CONCLUSION

In this study, we developed a milk yield-based indexing framework for identifying mastitis-associated changes in daily milk production. Index scores progressively decreased across the pre-diagnosis, diagnosis-centered, and post-diagnosis intervals in mastitis datasets, whereas no significant interval-dependent changes were observed in matched non-mastitis datasets. Classification performance depended on both the evaluation-window length and the evaluation end day. The highest F1-score in the development dataset was obtained using a 3-day relative-decline rule ending on day +7, while the independent test dataset showed the highest and most balanced performance from days +4 to +6. These findings suggest that sustained deviations from expected milk production patterns can provide a practical and standardized alert signal, although no single temporal configuration was optimal across datasets. Because the framework relies on routinely collected milk yield records, it may support noninvasive identification of cows requiring closer observation or confirmatory testing. Nevertheless, external validation using larger and more diverse farm populations and integration with additional sensor-derived or clinical indicators are needed to improve the generalizability, timeliness, and reliability of mastitis alerts.

## Figures and Tables

**Figure 1:**
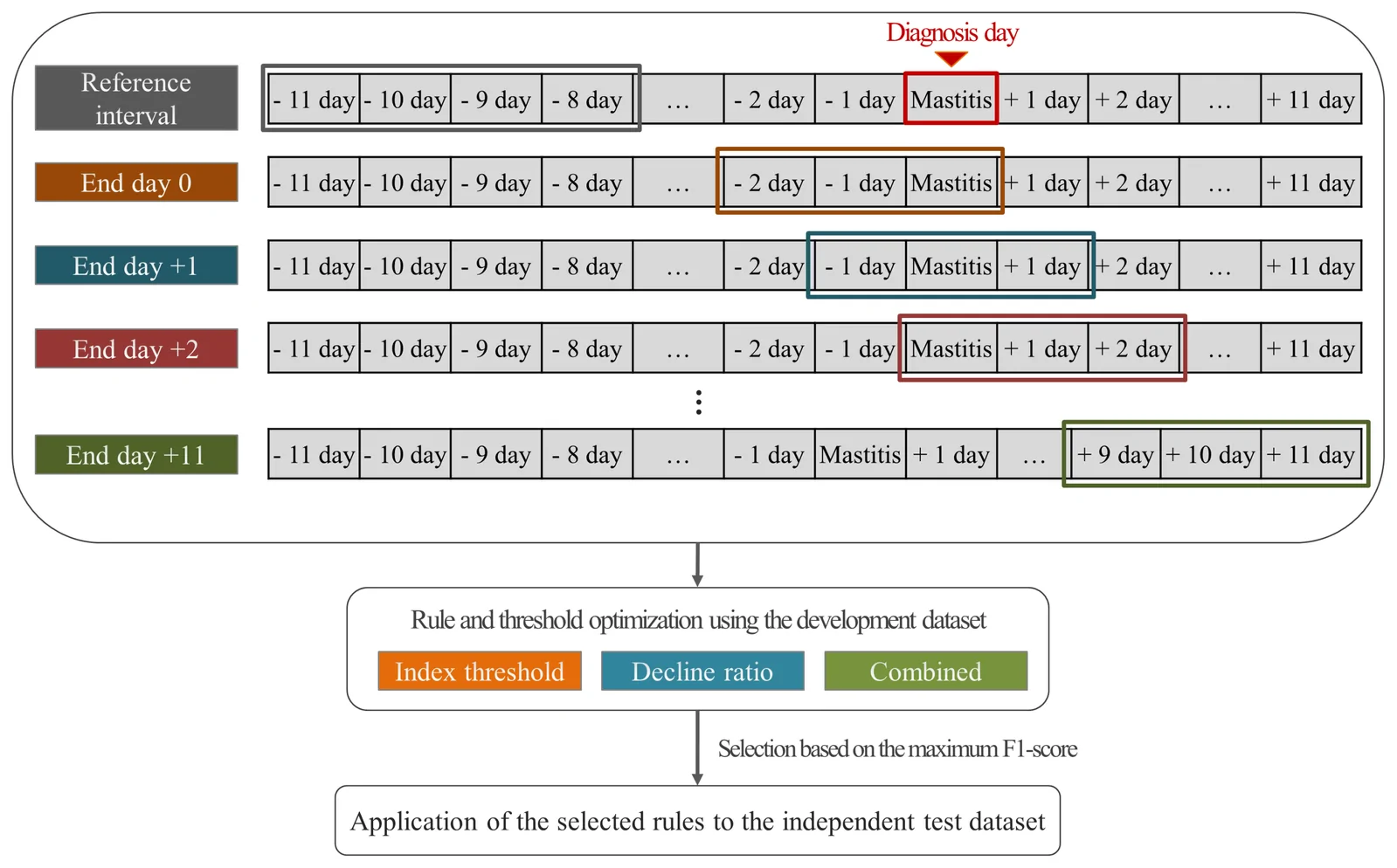
Analytical framework for the optimization and evaluation of the milk yield-based mastitis alert rules. A 3-day evaluation window is shown as an illustrative example. The reference index was calculated using records from day −11 to day −8 relative to the recorded mastitis diagnosis date. The 3-day evaluation window was sequentially shifted according to evaluation end days ranging from day 0 to day +11. At each evaluation end day, index-threshold, relative-decline, and combined rules were evaluated using the development dataset, and the rule and thresholds yielding the maximum F1-score were selected. The selected end-day-specific rules were subsequently applied to the independent test dataset without further optimization. The same procedure was performed for the 5- and 7-day evaluation windows.

**Figure 2:**
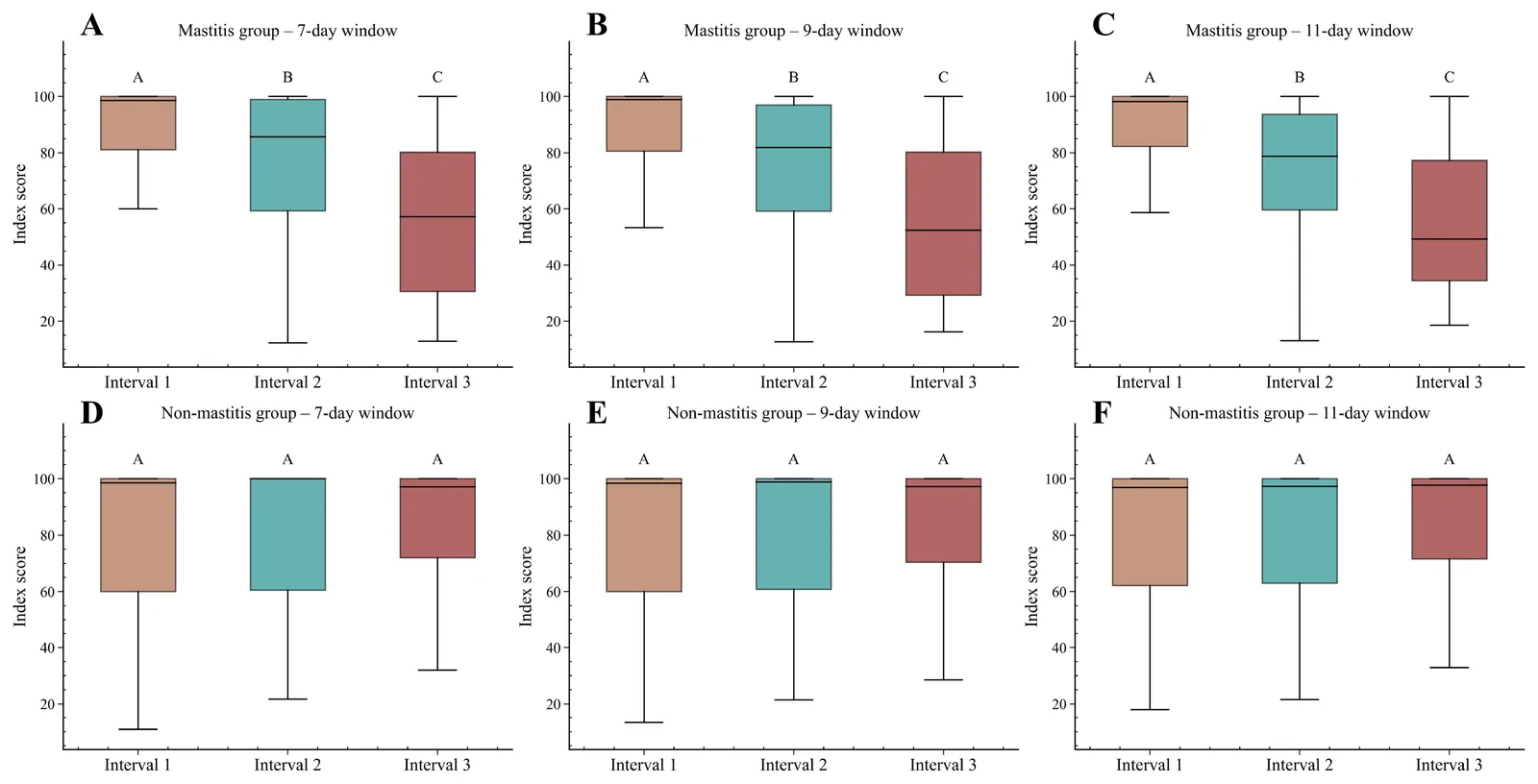
Comparison of interval-specific index scores between sequential evaluation intervals in mastitis and non-mastitis datasets using 7-, 9-, and 11-day windows in the development dataset. (A–C) present the distributions of interval mean index scores for mastitis datasets using 7-, 9-, and 11-day windows, respectively, whereas (D–F) present the corresponding distributions for non-mastitis datasets. Interval 1 represents the pre-diagnostic period, Interval 2 represents the diagnosis-centered period, and Interval 3 represents the post-diagnostic period. Boxes indicate the interquartile range, horizontal lines indicate medians, and whiskers indicate the data range excluding outliers. ^A–C^ Different letters above the boxes indicate significant pairwise differences among intervals within each window size according to Holm-adjusted Wilcoxon signed-rank tests (adjusted p<0.05).

**Figure 3:**
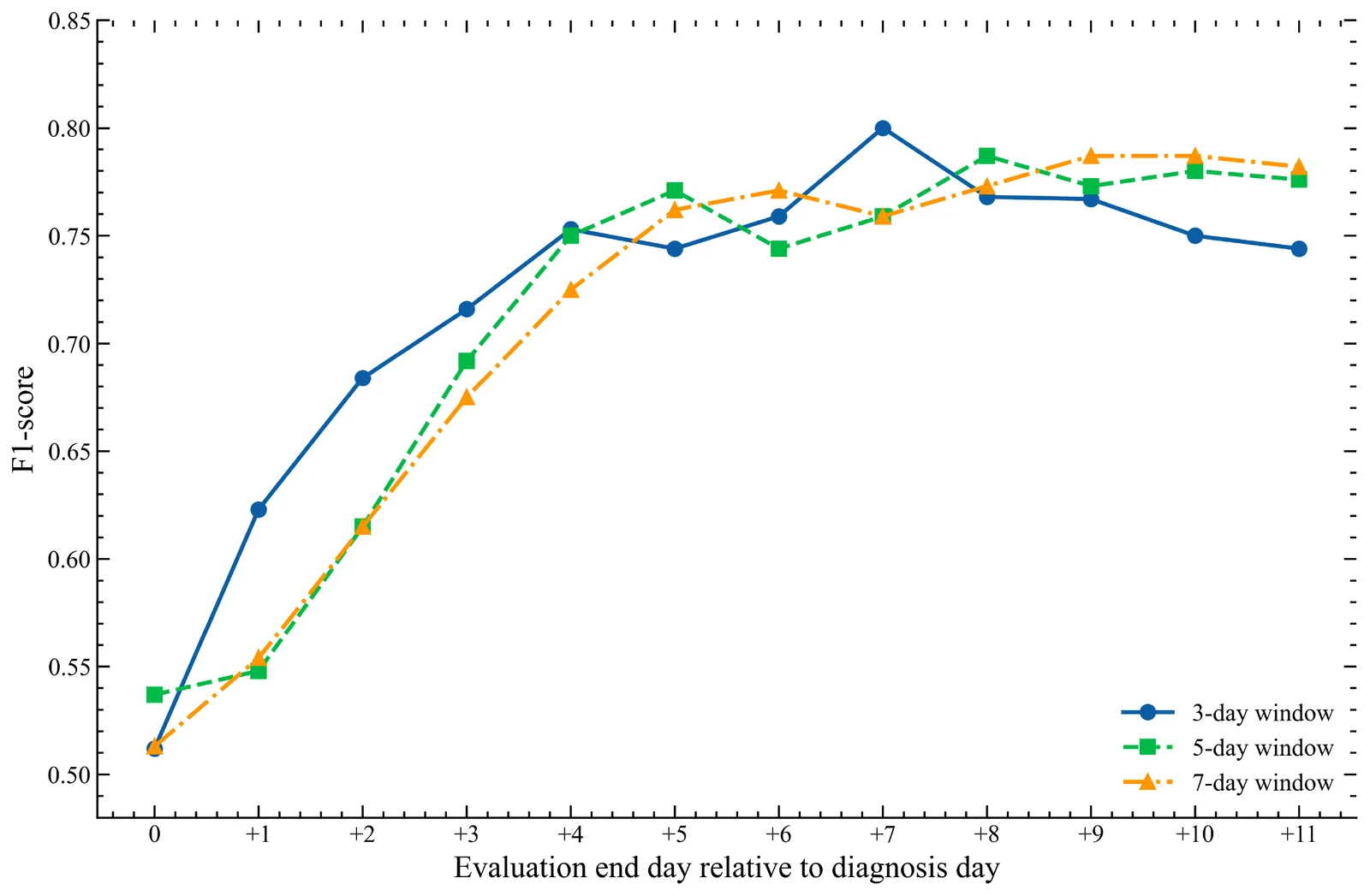
Changes in the F1-score of optimized milk yield-based mastitis alert rules according to evaluation end day relative to the recorded diagnosis date and evaluation-window length in the development dataset. F1-scores are presented for 3-, 5-, and 7-day evaluation windows at evaluation end days ranging from day 0 to day +11. Day 0 denotes the recorded mastitis diagnosis date, and positive values indicate the number of days after the recorded diagnosis date. For each combination of evaluation end day and window length, the alert rule and its associated threshold or thresholds were independently optimized in the development dataset based on the maximum F1-score. The evaluated rules included an index-threshold rule, a relative-decline rule, and a combined rule requiring both criteria to be satisfied.

**Table 1:** Distribution of mastitis datasets and diagnostic labels by farm in the raw data and final analysis data

Data stage	Farm no.	Mastitis lactation datasets1)	Unique mastitis events2)	Diagnostic labels			Multiple-label events3)

				CLOT	CMT	VO	
Raw	1	37	63	32	56	0	25
	2	44	62	2	0	60	0
	3	17	18	1	15	3	1
	4	21	34	34	0	2	2
	Total	119	177	69	71	65	28
Final analysis	1	21	21	12	17	0	8
	2	26	26	1	0	25	0
	3	2	2	0	1	1	0
	4	14	14	14	0	0	0
	Total	63	63	27	18	26	8

Farm 1 was located in Cheonan-si and used the Afimilk system; Farm 3 was located in Dangjin-si and used the Afimilk system; Farms 4 and 5 were located in Asan-si and used the DeLaval and BouMatic systems, respectively.

1) Mastitis lactation datasets indicate the number of identified lactations containing at least one recorded mastitis event.

2) A unique mastitis event was defined as a positive diagnostic record for the same cow on the same calendar date, irrespective of the number of diagnostic methods recorded.

3) Multiple-label events were defined as unique cow-date events with two or more concurrently recorded positive diagnostic labels.

CLOT, clot filter method; CMT, California mastitis test; VO, visual observation.

**Table 2:** Indexing criteria for milk yield evaluation based on the most recent 7-d period

Reduction rate1)		Confidence interval2)		Negative values3)	

Criterion (%)	Score	Criterion	Score	Criterion	Weight
<10	100	<1	−20	<4	1
10–20	90	2	−50	4	0.9
20–30	85	3	−60	5	0.8
30–40	80	4	−65	6	0.7
40–50	70	5	−70	7	0.6
>50	60	>6	−80		

1) Reduction rate = exponential smoothing value relative to the predicted value.

2) Confidence interval = number of data that fell below the lower bound of the confidence interval.

3) Negative values = number of consecutive negative values.

**Table 3:** Mean and standard deviation of interval-specific index scores according to group and window size in the development dataset

Group	Window size (d)	Interval 11) mean	Interval 22) mean	Interval 33) mean	Interval 11) SD	Interval 22) SD	Interval 33) SD
Mastitis	7	84.588	75.532	56.523	23.964	25.591	28.761
	9	85.490	74.167	54.952	22.883	25.017	27.782
	11	85.950	72.930	54.893	21.911	24.357	26.377
Non-mastitis	7	80.065	81.175	82.958	30.079	26.422	23.198
	9	80.093	81.384	82.788	29.021	25.745	23.317
	11	80.403	81.438	82.804	27.512	25.067	23.082

1) Interval 1, pre-diagnosis period.

2) Interval 2, diagnosis-centered period.

3) Interval 3, post-diagnosis period.

SD, standard deviation.

**Table 4:** Differences between interval mean index scores, mean absolute within-dataset differences, and variability of within-dataset differences according to group and window size in the development dataset

Group	Value		Difference between group means2)			Mean absolute individual difference			SD of individual differences		

	Interval1)		1 vs 2	1 vs 3	2 vs 3	1 vs 2	1 vs 3	2 vs 3	1 vs 2	1 vs 3	2 vs 3
Mastitis	Window size	7	−9.055	−28.065	−19.010	12.120	30.643	20.386	14.189	27.439	17.292
		9	−11.323	−30.538	−19.215	14.066	33.917	21.947	14.794	29.111	19.002
		11	−13.021	−31.058	−18.037	15.537	34.942	22.087	15.122	29.897	20.250
Non-mastitis		7	1.110	2.893	1.783	6.266	15.036	9.289	11.062	24.561	15.630
		9	1.290	2.694	1.404	6.679	14.856	9.611	11.438	25.043	16.047
		11	1.035	2.401	1.366	7.556	15.269	9.907	11.992	25.364	16.256

1) Interval 1 represents the pre-diagnosis period, Interval 2 represents the diagnosis-centered period, and Interval 3 represents the post-diagnosis period. For matched non-mastitis datasets, the corresponding reference date was used instead of a diagnosis date.

2) Differences were calculated as the mean index score of the second-listed interval minus that of the first-listed interval. Negative values indicate a lower index score in the second-listed interval.

SD, standard deviation.

**Table 5:** Classification performance and selected mastitis alert rules at key evaluation time points in the development dataset

End day1)	Window size (d)	Rule type2)	Index threshold	Decline threshold (%)	Accuracy	Sensitivity	Specificity	F1 Score
0	5	Index	94	-	0.568	0.500	0.636	0.537
+3	3	Ratio	-	5	0.739	0.659	0.818	0.716
+7 (best)	3	Ratio	-	5	0.796	0.818	0.773	0.800

A dash indicates that the corresponding threshold was not applicable.

1) Evaluation end day was defined relative to the recorded diagnosis date, with day 0 representing the diagnosis date and positive values indicating days after diagnosis. For matched non-mastitis datasets, the corresponding reference date was used.

2) The index rule classified a dataset using the mean index score within the evaluation window, whereas the ratio rule used the relative decline from the reference interval.

**Table 6:** Classification performance in the test dataset of mastitis alert rules optimized in the development dataset according to evaluation end day

End day1)	Window size (d)	Rule type2)	Index threshold	Decline threshold (%)	Accuracy	Sensitivity	Specificity	F1 Score
0	5	Index	94	-	0.526	0.632	0.421	0.571
+1	3	Ratio	-	5	0.632	0.474	0.790	0.563
+2	3	Ratio	-	5	0.658	0.526	0.790	0.606
+3	3	Ratio	-	5	0.763	0.737	0.790	0.757
+4	3	Ratio	-	5	0.790	0.790	0.790	0.790
+5	5	Combined	86	5	0.790	0.790	0.790	0.790
+6	7	Combined	83	5	0.790	0.790	0.790	0.790
+7	3	Ratio	-	5	0.737	0.790	0.684	0.750
+8	5	Ratio	-	5	0.737	0.790	0.684	0.750
+9	7	Ratio	-	5	0.737	0.790	0.684	0.750
+10	7	Ratio	-	5	0.737	0.790	0.684	0.750
+11	7	Combined	89	5	0.737	0.790	0.684	0.750

A dash indicates that the corresponding threshold was not applicable.

1) Evaluation end day was defined relative to the recorded diagnosis date, with day 0 representing the diagnosis date and positive values indicating days after diagnosis. For matched non-mastitis datasets, the corresponding reference date was used.

2) The index rule classified a dataset using the mean index score within the evaluation window, whereas the ratio rule used the relative decline from the reference interval. The combined rule required both the index and relative-decline criteria to be satisfied.

## Data Availability

Upon reasonable request, the datasets of this study can be available from the corresponding author.

## SUPPLEMENTARY MATERIAL

**Supplement 1**. Optimized classification performance for each combination of evaluation end day and window size in the development dataset.
