# Supplementary material for: Development and evaluation of a milk yield-based indexing framework for mastitis monitoring in dairy cows
Source: Anim Biosci. 2026 Sep 1;39(11):260447. doi: 10.5713/ab.260447 (PMC13740642; doi:10.5713/ab.260447)
Supplement: Supplementary file 1 [file ab-260447-Supplementary-1.pdf]

Supplement 1. Optimized classification performance for each combination of evaluation end day and window size in the development dataset

End day[1]	Window size (days)	Rule type[2]	Index threshold	Decline Threshold (%)	Accuracy	Sensitivity	Specificity	F1 Score
0	3	Index	94	-	0.546	0.477	0.614	0.512
0	5	Index	94	-	0.568	0.500	0.636	0.537
0	7	Index	90	-	0.568	0.455	0.682	0.513
1	3	Ratio	-	5	0.671	0.546	0.796	0.623
1	5	Ratio	-	5	0.625	0.455	0.796	0.548
1	7	Index	92	-	0.580	0.523	0.636	0.554
2	3	Ratio	-	5	0.716	0.614	0.818	0.684
2	5	Ratio	-	5	0.659	0.546	0.773	0.615
2	7	Ratio	-	5	0.659	0.546	0.773	0.615
3	3	Ratio	-	5	0.739	0.659	0.818	0.716
3	5	Ratio	-	7	0.727	0.614	0.841	0.692
3	7	Ratio	-	5	0.705	0.614	0.796	0.675
4	3	Ratio	-	5	0.761	0.727	0.796	0.753
4	5	Combined	86	5	0.773	0.682	0.864	0.750
4	7	Combined	90	5	0.750	0.659	0.841	0.725
5	3	Ratio	-	5	0.750	0.727	0.773	0.744
5	5	Combined	86	5	0.784	0.727	0.841	0.771
5	7	Combined	90	5	0.773	0.727	0.818	0.762
6	3	Ratio	-	5	0.761	0.750	0.773	0.759
6	5	Ratio	-	5	0.750	0.727	0.773	0.744
6	7	Combined	83	5	0.784	0.727	0.841	0.771
7	3	Ratio	-	5	0.796	0.818	0.773	0.800
7	5	Ratio	-	5	0.761	0.750	0.773	0.759
7	7	Ratio	-	5	0.761	0.750	0.773	0.759
8	3	Index	91	-	0.739	0.864	0.614	0.768
8	5	Ratio	-	5	0.784	0.796	0.773	0.787
8	7	Ratio	-	5	0.773	0.773	0.773	0.773
9	3	Ratio	-	5	0.773	0.750	0.796	0.767
9	5	Ratio	-	5	0.773	0.773	0.773	0.773
9	7	Ratio	-	5	0.784	0.796	0.773	0.787
10	3	Index	94	-	0.727	0.818	0.636	0.750
10	5	Index	95	-	0.750	0.886	0.614	0.780
10	7	Ratio	-	5	0.784	0.796	0.773	0.787
11	3	Ratio	-	5	0.750	0.727	0.773	0.744
11	5	Index	95	-	0.750	0.864	0.636	0.776
11	7	Combined	89	5	0.784	0.773	0.796	0.782

[1] Evaluation end day was defined relative to the recorded diagnosis date, with day 0 representing the diagnosis date and positive values indicating days after diagnosis. For matched non-mastitis datasets, the corresponding reference date was used.

[2] The index rule classified a dataset using the mean index score within the evaluation window, whereas the ratio rule used the relative decline from the reference interval. The combined rule required both the index and relative-decline criteria to be satisfied. A dash indicates that the corresponding threshold was not applicable.
